# Metagenomic detection of the complete coding regions of *Tanay virus* from mosquitoes (*Armigeres subalbatus*) in India

**DOI:** 10.1128/mra.00389-25

**Published:** 2025-09-12

**Authors:** Perumal Arumugam Desingu, Selvarayar Arunkumar, K. Nagarajan, G. Saikumar

**Affiliations:** 1 Department of Virus Epidemiology, Vector Dynamics & Public Health, Institute of Advanced Virology 681433 , Thiruvananthapuram, Kerala, India; 2 Department of Veterinary Parasitology, Madras Veterinary College, Tamil Nadu Veterinary and Animal Sciences University 29884, Chennai, Tamil Nadu, India; 3 Department of Veterinary Pathology, Madras Veterinary College, Tamil Nadu Veterinary and Animal Sciences University 29884, Chennai, Tamil Nadu, India; 4 Division of Pathology, Indian Veterinary Research Institute 30072, Izatnagar, Uttar Pradesh, India; Queens College Department of Biology, Queens, New York, USA

**Keywords:** *Tanay virus*

## Abstract

So far, the Tanay virus has only been detected in the Philippines and China. Here, we report that a complete coding region-wide virus with 3.7–16.7% nucleotide diversity to the Tanay viruses identified in China and 25.2% nucleotide diversity to those identified in the Philippines is circulating in mosquitoes (*Armigeres subalbatus*) in India.

## ANNOUNCEMENT

Currently, the Tanay virus (TANAV), which is classified within the genus *Sandewavirus* and is part of the newly proposed taxon *Negevirus*, has only been detected in the Philippines (1) and China (2, 3). In the Philippines, it was found in pools of *Culex* spp. and *Armigeres* spp. (1). In China, it was identified in *Culex tritaeniorhynchus*, *Culex quinquefasciatus*, and *Anopheles sinensis* (2, 3). In this study, we detected the complete coding regions of the TANAV from *Armigeres subalbatus* in India.

In this study, we collected *Armigeres subalbatus* mosquitoes from Nagercoil (Putheri Lake) located in the Kanyakumari District of Tamil Nadu, India. We pooled 20 mosquitoes to create a single sample by homogenizing them in PBS for virus metagenomic sequencing. From this pooled sample, we extracted RNA using TRI reagent following a standardized protocol (4). Library preparation was performed with the TruSeq Stranded Total RNA Kit (Illumina #15032618, Illumina #20020596), adhering to the manufacturer’s guidelines. Additionally, the insert size of the library was measured using TapeStation 4150 (Agilent) with D1000 screentapes (Agilent #5067–5582) following the manufacturer’s instructions. Finally, sequencing was conducted on Illumina NovaSeq 6000. In the paired-end sequencing, we generated 99,704,106 reads with a length of 150 bp. Subsequently, quality control and removal of low-quality and potential adapter sequences were conducted using FastQC (version 0.11.5) and Trimmomatic (5), respectively, following default parameters. The reads that passed quality control were filtered to virus-specific reads using the protein-based alignment method, DIAMOND (6), and the filtered reads were assembled *de novo* with metaSPAdes (7) using default settings. The assembled sequences were then analyzed with BLASTx and BLASTn against the National Center for Biotechnology Information (NCBI) RefSeq database to identify the viruses (8), and the virus-specific contig was aligned in the advanced genome aligner (AGA) (9) and the consensus variant caller GATK/BcfTools (10, 11), employing default parameters.

In this virus-metagenomic analysis, we identified the complete coding regions of the TANAV, which is 9,556 bp long with a guanine and cytosine content of 36.83%. This virus has three open reading frames (ORFs) as follows: ORF1 from 60 to 6,707 nucleotide positions with 2,215 amino acids, ORF2 from 6,731 to 8,515 nucleotide positions with 594 amino acids, and ORF3 from 8,645 to 9,292 nucleotide positions with 215 amino acids.

**Fig 1:**
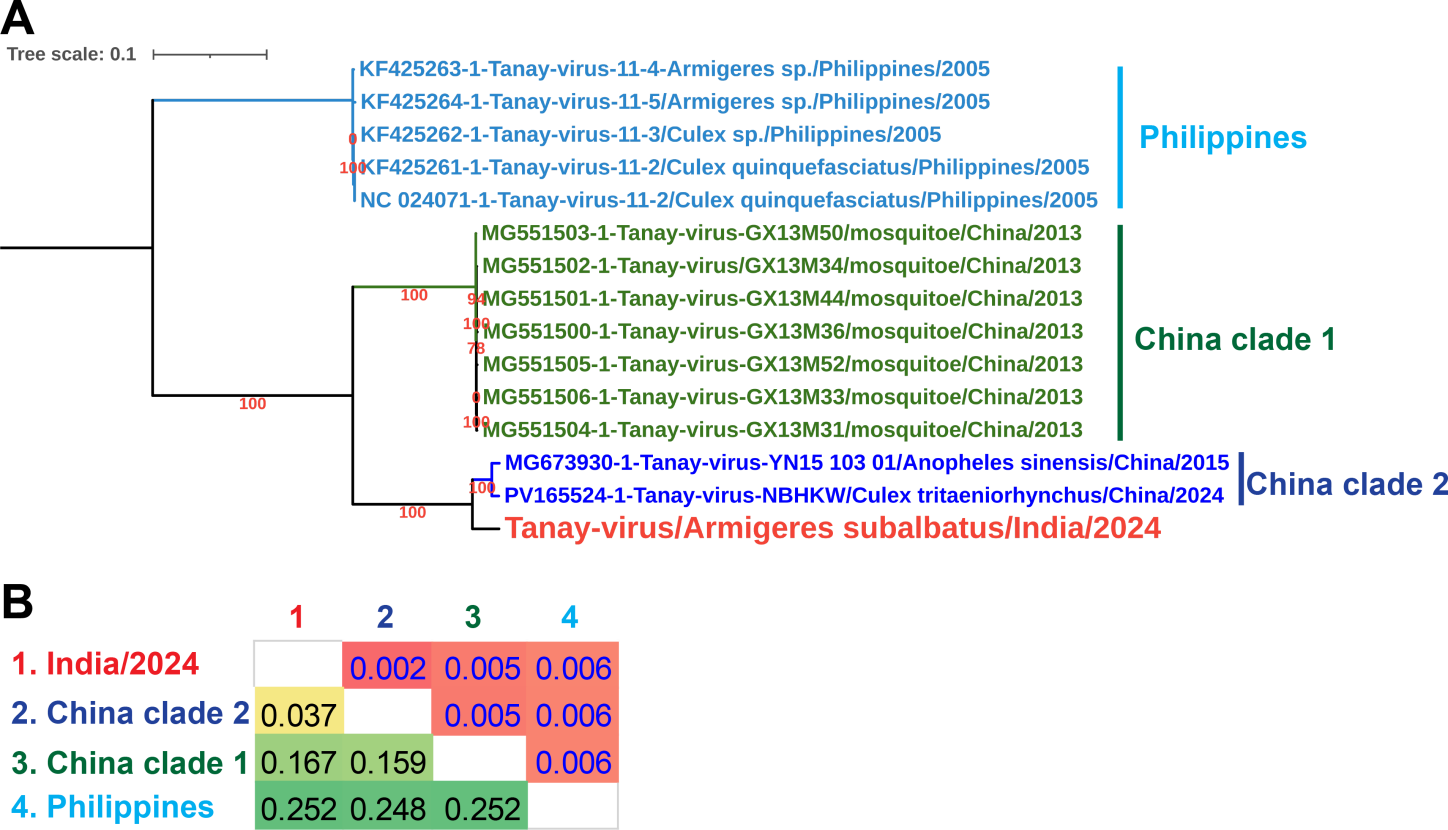
(**A**) The phylogenetic tree illustrates the various genotypes of the Tanay viruses. Complete genome sequences were aligned using MAFFT 7.407_1 (12, 13). Phylogenetic analysis was conducted using the GTR evolutionary model with PhyML 3.3_1 (14), and the tree was visualized using the Interactive Tree of Life (iTOL) version 5 (15). (**B**) The table presents the genetic diversity among the different genotypes of the Tanay virus based on the NBGMD analysis. Standard error estimates for the NBGMD analysis are indicated above the diagonal in the table. The nucleotide sequences were aligned with MAFFT 7.407_1 (12, 13), and these aligned sequences were used for the NBGMD analysis in MEGA7 (16) with the Kimura 2-parameter model applied, considering both transitions and transversions for substitutions. Gaps and missing data were removed using the pairwise deletion approach, and the analysis standard errors were calculated using bootstrap replicates (1,000 in total).

Our analysis has shown that the TANAV/India/2024 is closely related to a strain of the virus detected in *Anopheles sinensis* in China (MG673930.1) with the nucleotide identity of 95.76% in the NCBI BLAST analysis. Consequently, we categorized the TANAV into three distinct clades based on the complete genome sequence: the Philippines clade, China clade-1, and China clade-2 (Fig. 1A and B). The TANAV/India/2024 has displayed the genetic divergence of 3.7, 16.7, and 25.2% with China clade-2, China clade-1, and Philippines clade, respectively (Fig. 1B). Overall, our results indicate that the TANAV related to the China genotype 2 found in *Culex tritaeniorhynchus* and *A. sinensis* in China is circulating in *Armigeres subalbatus* in India.

## Data Availability

The generated RNA-Seq data have been submitted to the NCBI with the project ID PRJNA1233911 and nucleotide accession number PV260372.
